# CT colonography in detection of colorectal carcinoma

**DOI:** 10.2478/v10019-010-0012-1

**Published:** 2010-03-18

**Authors:** Amela Sofic, Serif Beslic, Igor Kocijancic, Nedzad Sehovic

**Affiliations:** 1 Institute of Radiology, Clinical Centre of University of Sarajevo, Sarajevo, Bosnia and Herzegovina; 2 Clinical Institute of Radiology, University Medical Centre Ljubljana, Ljubljana, Slovenia

**Keywords:** CT colonography, barium enema, colonoscopy, colorectal polyp, colorectal carcinoma

## Abstract

**Background:**

Diagnostic methods used in screening and detecting colorectal carcinoma are digitorectal examination, faecal occult blood testing, sigmoidoscopy, DNA stool analysis, barium enema, colonoscopy, and as of recently CT colonography. The aim of this study was to establish diagnostic accuracy and comfort of CT colonography compared to colonoscopy and barium enema.

**Patients and methods.:**

We included 231 patients in the prospective study. For all patients CT colonography and barium enema followed by colonoscopy were performed. After the procedures a comfort assessment was done in all patients. Diagnostic positive results were verified by the pato-histological examination. Sensitivity, specificity, positive predicative value (PPV) and negative predicative value (NPV) were calculated for each procedure.

**Results:**

With CT colonography, barium enema and colonoscopy 95 lesions were found, 56 (59%) of them were tumours and 39 (41%) were polyps. Among polyps pato-histology revealed 34 adenomas, 3 tubulovillous adenomas and 2 lipomas, among tumours there were 55 adenocarcinomas and 1 lymphoma. Results showed CT colonography sensitivity to polyps to be 89.7%, barium enema 48.7%, and colonoscopy 94.9%. Sensitivity to tumours of CT colonography and colonoscopy was 100% and of barium enema 94.6%. Specificities and PPV were 100% in all procedures. The comfort assessment showed CT colonography as the far most comfortable out of three procedures.

## Introduction

Colorectal carcinoma (CRC) is the second leading cause of illness and the third leading cause of death in Western countries.^1^ Pato-histologically (PH) CRC is most commonly adenocarcinoma in 98% of cases. CRC starts as a polyp, representing precursor of CRC. Consumption of meat and animal fats, physical inactivity, smoking and consumption of alcohol increase the risk for CRC.

Prevention and screening of CRC are very complex and depend on financial and organizational capacities of health institutions where they are performed. There are several basic tests applied in the screening of CRC: digitorectal examination, faecal occult blood testing (FBOT), sigmoidoscopy, colonoscopy, barium enema, DNA stool analysis and recently CT colonography (CTC).^2^,^3^

We conducted this comparative study to establish the diagnostic accuracy and comfort of CTC comparing with C and barium enema.

## Patients and methods

Of 231 patients included in the study 106 (47%) were males and 125 (53%) were females. The average age of patients was 57.9 years (SD ± 11.3y, range 23–83y). Only patients with suspected symptoms of CRC were included with the history of blood in the stool, anaemia, constipation, and changes in the stool or positive FBOT test.

In all patients CT colonography, barium enema and colonoscopy were performed. Positive diagnostic findings were correlated with PH results of biopsies taken during colonoscopy. Two hundred and twenty-seven patients were included in the statistical analysis; four patients were excluded due to undetermined PH results.

An identical protocol for cleansing the bowels (Dulcolax® tablets and suppositories, as well as Coloclens® syrup) was performed before the commencement of each of three procedures. The CTC procedure was performed after the air had been insufflated in the cleansed colorectal region until an optimal extension, with an intravenous application of spasmolytics. Patients with intraluminal residual content or suboptimal distension of the bowels were excluded from the study so that reliable images could be achieved. CT scanning was performed on 4 slice MDCT (Volume zoom Siemens, Erlangen, Germany) equipment in the prone and supine position of the patient. 2D and 3D reconstructions were performed on the «Syngo» software work station.

Double contrast barium enema was performed on an X-ray diascopic equipment (Practix 100, Philips, Aidhoven The Netherlands). The colonoscopy procedure was performed by a gastroenterologist on Videocolonoscopic device (CF Q-165 L Olympus» Tokyo Japan). PH examination was done on the tissue obtained by polypectomy or tumour sample that was taken either during an endoscopic examination or a surgical procedure.

In relation to PH findings sensitivity and specificity as well as PPV and NPV, using Kappa statistical method for all three procedures were calculated. All hypotheses were tested for the statistical significance of p <0.05 value. Confidence intervals (CI) were also presented. Patients self evaluated comfort of all three procedures as being comfort, less comfort or discomfort.

## Results

The histological examination was conclusive in 227 patients. There were 39 benign lesions in 31 patients and 56 malignant lesions in 56 patients. Benign lesions were present among females in 22 cases (56%), and in males in 17 cases (44%). In male patients tumours were found in 30 cases (54%), and in the females in 26 (46%). Age distribution of the patients regarding benign lesions and tumours is presented in Table 1 (Table 1). The most common symptoms in the case of polyps were: bowel disturbances in 14 cases, constipation in 14 cases, blood in the stool in 7 cases, followed by anaemia and abdominal pain each in 1 case. In case of tumours, most commonly reported symptoms were: blood in the stool in 35 cases, anaemia in 11 cases and constipation in 10 cases.

Most polyps were detected in colon descedens, followed by rectum, colon transversum, caecum and colon ascendens. The most frequent localization of carcinoma was rectum in 27 cases followed by sigmoid part of colon in 13 cases while the descedent part of colon in 5 cases. In the remaining nine cases, carcinoma was found in colon ascedens, transversum and caecum, three cases in each of these localisations. In one case carcinoma was located on hepatic flexure whilst a single case of lymphoma was located on Valvula Bauchini.

**FIGURE 1:**
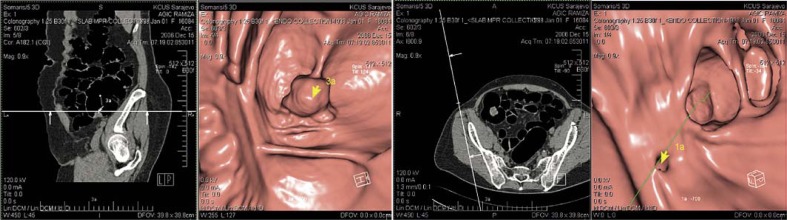
Polypous adenoma of cecal region in a 55 years old female patient, obtained by our CTC evaluation.

With the CTC procedure the size of benign lesions (polyps) detected was: less than 6 mm in 2 cases, 6 to 10 mm in 15 cases and larger than 10 mm in 18 cases. The size of carcinomas detected by CTC was more than 10 mm in all 56 cases (Figure 1).

Barium enema detected benign lesions between 6 and 10 mm in 2 cases and in 17 cases larger than 10 mm. This procedure did not detect any polyps smaller than 6 mm. With BE 53 carcinomas were found, all of them were larger than 10 mm.

Colonoscopy detected benign lesions smaller than 6 mm in 6 cases, 6 to 10 mm in 9 cases and larger than 10 mm in 22 cases. The size of carcinomas detected by colonoscopy was larger than 10 mm in all 56 cases.

**FIGURE 2:**
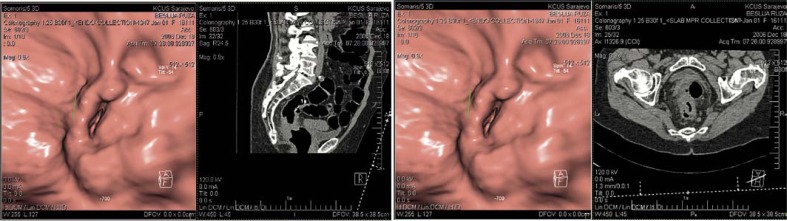
Adenocarcinoma in the middle third part of the rectum with infiltration of mesorectal fat tissue and reactive lymph nodes, obtained by our CTC evaluation.

Amongst polyps, there were 34 adenomas, followed by tubuloviluous adenomas in three cases and lipomas in three cases. According to PH analysis adenocarcinoma was far most common (in 98% of cases, n=55), since there was only a single case of lymphoma. (2%). (Figure 2). In all 231 patients CTC side findings were found in 25 cases and extracolic extension in 36 cases.

Sensitivity, specificity and PPV for all three procedures are presented in Table 2. We obtained statistically significant results validating CTC procedure regarding sensitivity and specificity on polyps and tumours, which are approximately identical in comparison with colonoscopy, and significantly above the method of barium enema. It is important to point out that the CTC method missed to locate 4 polyps which were found by colonoscopy, but did not miss any tumours. Out of 22 cases which were missed by barium enema, all 22 were located by colonoscopy, and 19 by CTC. 3 of those cases were carcinoma and all were diagnosed by both colonoscopy and CTC. Colonoscopy did not miss any carcinoma; however it missed two polyps located by CTC. Both were later confirmed by colonoscopy and PH; however, it did not miss any tumours. To evaluate staging of carcinoma, we used Dukes method of clinical staging and achieved conformity in 96.4% of cases in comparison with the post-surgical oncology staging.

In a survey of all examinees in our research, the CTC procedure was assessed as the most comfortable in comparison with the barium enema and colonoscopy; all 231 patients assessed the CTC procedure as comfort. Barium enema was assessed as a less comfort procedure by 224 of patients, and as discomfort by 7 patients. CC was assessed as the least comfort procedure by 224 patients, assessed it as discomfort, whilst 7 patients assessed it as less comfort.

## Discussion

In recent years there has been an extremely rapid development of CT due to the development of CT multislice technology. Its more frequent use in detecting CRC is also due to the fact that it has not yet been established an optimal procedure regarding comfort and high reliability in detection of colorectal lesions.^1^

The CTC could become an important method in CRC and polyps screening due to its efficacy, cost-effectiveness and because it is an ultra-low dose radiation technique.^4^,^5^ The more recent method is MR imaging. However, it is usually used in the diagnostic of colorectal lesions and not in the screening proceeding.^6^ The most significant advantage of CTC is that it can detect the extraluminal tumour extension, which is not possible by other procedures.^7^ It is extremely important for discovering the extent of the disease and enabling the proper choice of the treatment. On that way we can influence on better surviving and quality of life of our patients.^8^,^9^ Regarding comfort, the CTC procedure is undoubtedly in advance compared to other two procedures. It is also much safer, although the colorectal injuries during barium enema are very rare.^10^ In our research the comfort of the procedure was assessed as being 100%. Gluecker published that 72% and Svenson stated that 82% of patients would rather have a CTC than any of the other two procedures.^11^,^12^

There are many reports regarding detecting benign and malignant colorectal lesions in the literature. Winawer published the lowest result regarding sensitivity of barium enema in detecting polyps to be 48%.^13^ Smith reported sensitivity of barium enema in detecting tumours as 83% and of colonoscopy as 97.5%.^14^ Hara stated that sensitivity of CTC for polyps larger than 1 cm was 75%, or 85% in a follow up study.^15^ Fletcher reported that sensitivity of CTC for polyps larger than 1 cm was 85%.^16^ Gennen published that sensitivity of barium enema regarding carcinoma is in the range of 85–95%, and that sensitivity in detecting polyps smaller than 1 cm is between 50–80%.^17^ Johnson published that sensitivity of CTC to polyps larger than 1 cm was 81%, and of barium enema of 45%. For those smaller than 1 cm, sensitivity of CTC was 72%, compared to barium enema which was 44%. Specificities of CTC were 96–99%, compared to 99–100% of barium enema.^18^ Cotton’s multicentric study included 600 participants and showed that sensitivity of CTC to lesions smaller than 6 mm was 39%, to those larger than 1 cm was 96%.^19^ Macari reported 100% sensitivity of CTC regarding polyps larger than 1 cm, and 52.9% regarding those between 6–9 mm.^20^ Iacanconne found 100% sensitivity of CTC regarding polyps of 1 cm and larger, and 86% for those smaller than 6 mm, which is slightly more compared to colonoscopy, which was 84%.^21^ Mulhal reported 48% sensitivity of CTC regarding polyps smaller than 6 mm; 70% for those between 6–9 mm; and 85% for those larger than 9 mm.^22^ Ramjii reported sensitivity of 71–93% for polyps larger than 1 cm, 55–71% for those between 5 and 9 mm, and 39% for those smaller than 6 mm.^23^ In 2008, Johnson acquired 90%sensitivity of CTC for polyps larger than 9 mm.^24^

In our research CTC was equally sensitive (100%) in detection of CRC lesions as colonoscopy and much better than barium enema (94.6%). The CTC demonstrated similar sensitivity in detecting polyps larger than 1 cm (89.7%) compared to the colonoscopy (94.9%), and better sensitivity compared to barium enema (sensitivity of 48.7%). The CTC is very efficient in pain-intolerant patients and in cases of tumours causing obstruction, dolichocolons, spasms, and other reasons preventing the colonoscope to reach the caecum. The CTC is suitable for screening and staging of tumours, as well as for obtaining unexpected findings on other abdominal or pelvic organs. In the detection of lesions smaller than 5 mm, colonoscopy showed to be better in regard to other two methods.

Having considered all results of our study and having compared all three procedures, we have obtained statistically significant differences regarding sensitivity and specificity of CTC regarding polyps and tumours. These results are quite similar to those compared to colonoscopy, but much more advanced compared to the barium enema. We could state here that our results regarding sensitivity of CT colonography are much better compared with the results of initial studies published in the early nineties in the world, and are quite close to the results of the latest studies published at the beginning of this century.

## Figures and Tables

**TABLE 1:** Age distribution of patients with positive hystologic findings

**Age**	**Polyps**		**Tumours**	
	**n**	**%**	**n**	**%**
20–30	4	10.26%	0	0.00%
31–40	5	12.82%	1	1.79%
41–50	10	25.64%	4	7.14%
51–60	10	25.64%	19	33.93%
61–70	9	23.08%	11	19.64%
71-...	1	2.56%	21	37.50%

**Total**	**39**	**100.00%**	**56**	**100.00%**

**TABLE 2:** Comparison of results regarding all three methods

	**Polyps**			**Tumours**			**All**		
	**CTC**	**BE**	**CC**	**CTC**	**BE**	**CC**	**CTC**	**BE**	CC
**Sensitivity**	89.7%	48.7%	94.9%	100.0%	94.6%	100.0%	95.8%	75.8%	97.9%
**Specificitiy and PPV**	100.0%	100.0%	100.0%	100.0%	100.0%	100.0%	100.0%	100.0%	100.0%
